# Malignant adenomyoepithelioma of the breast: A case report

**DOI:** 10.1016/j.ijscr.2020.05.061

**Published:** 2020-05-30

**Authors:** Eisa A. Lari, Ali A. Lari, Talal Alsaeed

**Affiliations:** General Surgery Department, Jaber Al-Ahmad Hospital, Ministry of Health, Kuwait

**Keywords:** Breast, Malignant, Adenomyoepithilioma, Mastectomy, Wide local excision

## Abstract

**Introduction:**

Malignant breast adenomyoepithelioma (AME) is a rare subtype of breast cancer. AME is mostly a benign disease that uncommonly undergoes malignant transformation.

**Presentation of case:**

Here we present a case of a young, previously healthy female who initially presented with a painless breast lump. The patient underwent wide local excision for atypical cells with squamous metaplasia by core biopsy, but final histopathology showed AME with carcinoma. Thus, a mastectomy and a sentinel lymph node biopsy was undertaken. The patient had an uneventful recovery and no recurrence after the second surgery.

**Discussion and conclusion:**

Malignant transformation of adenomyoepithelioma is reported in a small number of cases. Benign AME may be treated with wide local excision as recurrence is rare locally. Whereas malignant AME tends to be treated with simple mastectomy with or without lymph node biopsy.

## Highlights

• Malignant breast adenomyoepithelioma (AME), rare subtype of cancer that usually benign.
• Mastectomy and sentinel lymph node biopsy with no further recurrence.
• Inner epithelial cells revealed positivity for CK5/6.
• Myoepithelial layers were positive for P63, CK5/6, SMA and S100 protein.
• Metastases chance of 30–40% with malignant adenomyoepithelioma, generally haematogenous route. Whereas metastases to the axillary lymph nodes is rare.

## 1 Introduction

Adenomyoepithelioma (AME) was first described in the 1970s as a neoplasm consisting of both luminal and myoepithelial cells [1]. This type of neoplasm exhibits a spectrum of morphology and display biphasic appearance in different areas of the tumor. Thus, making it diagnostically challenging by core biopsy due to its heterogeneity [2]. It has been reported to occur between the third and ninth decade, but more commonly in the 5th and 6th decade [3].

Macroscopically, AME are usually well circumscribed, solid, unencapsulated and may show focal cystic changes [4]. Microscopically, malignant AME is distinguished from benign adeno-myoepithelioma by the presence of nuclear atypia, coarse chromatin, prominent nucleoli, necrosis and increased mitotic rate [5].

This case report has been reported in line with the SCARE criteria [14].

## 2 Case report

A 39-year-old female presented with a lump in her left breast with no other complaint. A mammogram showed an ill-defined irregular mass in UOQ with no suspicious microcalcification, an ultrasound showed a hypoechoic mass at 9 o’clock 2 × 1.5 cm in size. A core needle biopsy was performed, which showed atypical cells with squamous metaplasia and sclerosing lesion with atypia. Thus, wide local excision was performed and final histopathology showed AME with carcinoma and positive margin. Subsequently, she underwent a mastectomy and SLNB. The patient had an uneventful recovery.

**Fig. 1:**
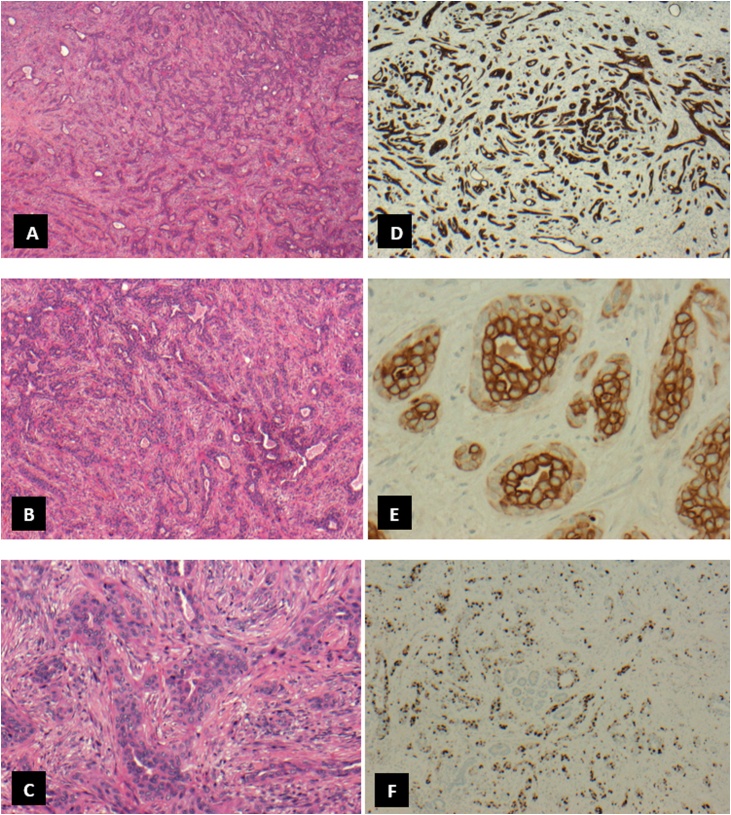
Histological features of adenomyoepithelioma with carcinoma exhibiting biphasic pattern of tubules lined by luminal epithelial and outer myoepithelial layers showing nuclear pleomorphism and mitosis (H&E in A, B and C). Immunohistochemical staining for P63 (D) and Ck5/6 (E) highlight myoepithelial layers while KI 67 (F) is about 30%.

Final histopathology revealed AME with carcinoma. Macroscopically, the mass presented as grayish white irregular mass approximately 3 × 2 × 1 cm in size. Microscopically, it showed biphasic tubular proliferation lined by inner epithelial cells positive for CK8/18 and negative for P63, SMA and S100 protein and outer myoepithelial layers positive for P63, CK5/6, SMA and S100 protein. The inner epithelial cells revealed strong positivity for CK5/6 as well. Both layers harbored atypical nuclei with obvious pleomorphism, hyperchromasia and frequent mitosis. Overall KI67 PI reached 30%. The proliferation infiltrated the adjacent non-neoplastic mammary tissue with occasional satellites at the periphery. The tumor was triple negative for ER, PR and ER (Fig. 1).

## 3 Discussion

Adenomyoepithelioma tend to be benign in nature, but malignant transformation is reported in a small number of cases. This is characterized by increase in mitotic rate, necrosis, atypia and prominent infiltrative growth [6,7]. Usually, malignant transformation occurs in one cellular component either epithelial or myoepithelial. However, malignant transformation in both cellular component is extremely rare [8,9].

Benign AME can be treated with wide local excision as it’s rare for it to recur locally. In contrast, the malignant type is more likely to recur locally and has a 30–40% chance of metastases, commonly through heamatogenous route to the lungs, brain, thyroid and chest wall [10]. However, metastases to axillary lymph node is rare [11,12].

Our case shows the difficulty in obtaining a clear diagnosis on core biopsy, and how this type of neoplasm should be considered in both benign and malignant lesions.

## 4 Conclusion

Surgical treatment of AME necessitates wide local excision with negative margins. However, patients with close or incomplete margin will need a simple mastectomy or re-excision to negative margin. As metastasis to axillary lymph nodes is rare some authors have recommended mastectomy with sentinel lymph node biopsy as the treatment of choice [7,9,10,13].

In conclusion, ideal surgical management has not been established yet due to its rarity.

## Sources of funding for your research

The research did not receive any funding.

## Ethical approval

The study is exempt from ethical approval – observational case report.

## Consent

Written consent was acquired from the patient.

## Author contribution

Eisa Lari – Study concept, data collection, **Guarantor**

Ali Lari – Data analysis, manuscript draft, review

Talal Alsaeed – Manuscript review, submission and final review

## Registration of research studies

NA.

## Guarantor

Dr. Eisa Lari.

Eisalari@gmail.com

## Provenance and peer review

Editorially reviewed, not externally peer-reviewed.

## Declaration of Competing Interest

The authors declare no conflict of interest.
